# Nicotine: evolution, dependence, and the path to total quit: redefining cessation in a changing nicotine landscape

**DOI:** 10.3389/fpubh.2026.1784188

**Published:** 2026-09-29

**Authors:** Maciej L. Goniewicz, Alex Bobak, Darush Attar-Zadeh, Matthias Raspe

**Affiliations:** 1 Department of Human Behavior, Division of Cancer Prevention and Population Sciences, Roswell Park Comprehensive Cancer Center, Buffalo, NY, United States; 2 Smoking Cessation Specialist, NHS, Surrey, United Kingdom; 3 Temple Consultancy Ltd., London, United Kingdom; 4 Department of Infectious Diseases and Respiratory Medicine, Charité - Universitätsmedizin Berlin, Berlin, Germany

**Keywords:** harm reduction, nicotine cessation, nicotine dependence, nicotine replacement, smoking cessation

## Abstract

**Background:**

The nicotine marketplace has expanded beyond combustible cigarettes to include electronic nicotine delivery systems (ENDS), heated tobacco products (HTPs), nicotine pouches, and licensed medicinal nicotine products. This diversification has altered patterns of exposure, dependence, and cessation, while complicating the meaning of “quitting” in public health and clinical practice.

**Objective:**

To review the evolving evidence on nicotine dependence across product categories and to examine whether smoking cessation alone remains an adequate endpoint in a landscape where nicotine use may persist beyond combustible tobacco.

**Methods:**

This narrative review synthesises evidence from neurobiology, epidemiology, clinical cessation research, and public health literature. Priority was given to systematic reviews, meta-analyses, clinical guidelines, and population-level data relevant to nicotine dependence, product risk, and cessation pathways.

**Findings:**

Nicotine dependence is shaped by pharmacology, delivery kinetics, behavioural conditioning, and product design. While non-combustible nicotine products may reduce exposure to smoking-related toxicants for people who switch completely from smoking, they may also sustain nicotine dependence, support dual use, and create new pathways to initiation among individuals who might otherwise have remained nicotine-free. Current cessation frameworks often focus on smoking abstinence without fully addressing continued nicotine dependence, particularly among long-term users of ENDS, HTPs, nicotine pouches, and other non-combustible products.

**Conclusion:**

In a changing nicotine landscape, cessation frameworks should distinguish more clearly between smoking cessation and nicotine cessation, support users across all nicotine product types, and re-establish complete freedom from nicotine dependence (*Total Quit*) as the optimal endpoint where achievable.

## Introduction

Nicotine dependence has historically been understood through the lens of cigarette smoking. For much of the tobacco control era, smoking cessation was functionally similar to ending nicotine use because combustible cigarettes were the dominant nicotine delivery system. That situation no longer holds. The current nicotine marketplace includes Electronic Nicotine Delivery Systems (ENDS), Heated Tobacco Products (HTPs), oral nicotine products such as pouches, and licensed medicinal nicotine products,^1^ each with distinct pharmacological, behavioural, and regulatory characteristics (1–3).

This shift has important implications for both clinical care and public health. For some smokers, non-combustible nicotine products may reduce exposure to combustion-related toxicants when they fully replace cigarettes, and this has informed harm reduction approaches in tobacco control (4, 5). However, reduced exposure is not the same as resolution of nicotine dependence. In many cases, nicotine use continues through alternative products, often over extended periods. In some populations, particularly young adults, these products have also introduced new pathways for nicotine initiation among individuals with no prior history of smoking^2^ (6–8).

At the same time, the language of cessation has become less precise. In many public health and clinical contexts, “quitting” now refers primarily to smoking abstinence, even when nicotine use continues. This may undermine the significance of ongoing dependence and leave some users without a clear cessation endpoint or access to structured support (3, 9, 10).

This narrative review examines the evidence on nicotine dependence across combustible and non-combustible products, including addiction mechanisms, comparative health risks, use patterns, and cessation strategies. It advances the position that while harm reduction may be appropriate for selected smokers as part of a stepwise pathway away from combustible tobacco, complete nicotine abstinence should remain the preferred long-term clinical and public health endpoint in order to achieve long-term freedom from addiction and eliminate any residual risks associated with ongoing use, whatever the form. In that sense, “total quit” is not intended as a slogan, but as a position statement: that freedom from nicotine dependence in all its forms remains the clearest endpoint where achievable.

## Methods and scope of review

This paper is a narrative review. Its purpose is not to provide a fully systematic or exhaustive synthesis of all available evidence, but to integrate key findings relevant to nicotine dependence, product risk, cessation, and clinical practice in the context of a rapidly evolving nicotine marketplace.

Targeted literature searches were undertaken using PubMed/MEDLINE and Google Scholar, drawing on foundational neurobiological work, contemporary clinical trials, systematic reviews, meta-analyses, national surveillance data, and major policy or consensus reports published through early 2025. Priority was given to Cochrane reviews, guideline documents, and reports from organisations such as the World Health Organization, the U. S. Surgeon General, the UK National Institute for Health and Care Excellence (NICE), and relevant UK surveillance sources, including the Office for National Statistics and the Smoking Toolkit Study. Information regarding product manufacturing and regulation was sourced from the European Tobacco Products Directive (2014/40/EU) and relevant patent documentation.

The literature was synthesised around four broad themes: (1) nicotine pharmacology and the mechanisms of dependence; (2) product characteristics and comparative risk; (3) patterns of use and public health implications; and (4) cessation pathways, including both smoking cessation and broader nicotine cessation. In areas where evidence remains limited, particularly for newer non-combustible products and long-term outcomes, interpretation was guided by the strength, consistency, and relevance of the available evidence.

Sources were selected based on their relevance to public health policy and clinical practice. Peer-reviewed data were prioritised to minimise bias.

The conclusions of this narrative review should be read considering an evidence base that remains incomplete and continues to evolve, especially regarding newer product categories, long-term patterns of use, and non-smoking nicotine dependence.

## Nicotine dependence in the modern product landscape

Nicotine is the principal constituent responsible for the dependence-producing effects of tobacco products and other nicotine delivery systems. It acts mainly through nicotinic acetylcholine receptors, particularly the α4β2 receptor subtype, promoting dopamine release within reward pathways and contributing to reinforcement, arousal, mood modulation, and repeated use (11, 12). Dependence is influenced not only by the pharmacology of nicotine itself, but also by the speed and efficiency with which it is delivered. Rapid systemic delivery, especially via inhalation, increases reinforcement and supports the development of repeated, highly conditioned use patterns (11, 13).

Nicotine dependence is further shaped by neuroadaptation and behavioural conditioning. Repeated exposure leads to receptor desensitisation and compensatory changes in receptor density, while routines, emotions, and environmental cues become strongly associated with use. These conditioned associations may persist beyond the acute withdrawal phase and remain a major contributor to relapse (14, 15). Individual risk is also modified by developmental, genetic, and metabolic factors, including variation in CYP2A6 function and nicotinic receptor subunits, as well as psychosocial influences such as stress, early experimentation, and socioeconomic disadvantage (16–18).

In the modern product landscape, dependence risk is also shaped by product design. Inhaled products such as ENDS and HTPs can deliver nicotine rapidly, with pharmacokinetic profiles that in some cases approach those of cigarettes, while some disposable ENDS and high -strength nicotine pouches may deliver high nicotine doses that reinforce repeated use (19–22). Features such as flavouring, ease of concealment, smoking-like rituals, and frequent access may further support habitual use and escalation (23).

A clinically relevant consequence of this diversified marketplace is transferred dependence, the persistence of nicotine dependence after cessation of combustible tobacco, where nicotine use continues through another product such as an ENDS, HTP, pouch, or other non-combustible format (Figure 1). Among ex-smokers using these products, product characteristics such as high nicotine concentration, rapid delivery, and (in the case of ENDS and HTPs) mimicry of smoking behaviours contribute to continued use. For some individuals, this may represent a clinically acceptable intermediate outcome if it reduces exposure to the harms of smoking. For others, however, it becomes an unintended long-term endpoint, particularly when the original goal was to stop nicotine use altogether (9, 24). Unlike medicinal nicotine products, non-combustible nicotine products typically lack formal guidance for reducing or discontinuing use, and transferred dependence to such products is rarely managed within a structured clinical framework (25). Most are positioned as recreational products rather than therapeutic aids, and behavioural support is seldom offered alongside them. By contrast, clinical best practice for medicinal nicotine products emphasises adequate initial dosing and sufficient duration to relieve cravings and reduce relapse risk (26).

**Figure 1:**
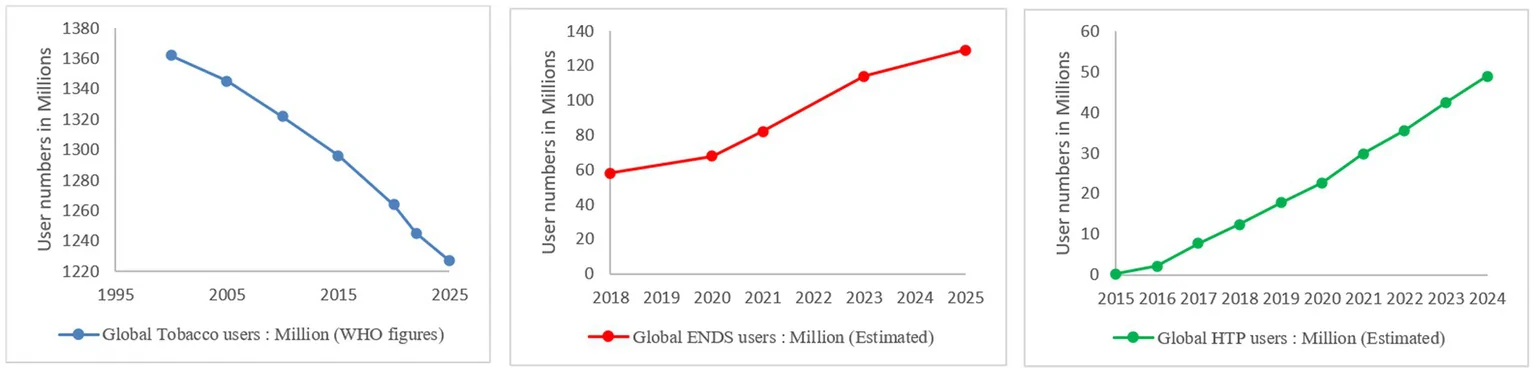
Trends in global estimates (in millions) of cigarette users, ENDS users, and HTP users based on the following sources: Cigarette users: WHO global report on trends in prevalence of tobacco use 2000–2030. Geneva: World Health Organization; 2024. ENDS users: Jerzyński, T., Mzhavanadze, G. and Stimson, G. V., 2026. Estimation of the global number of nicotine vapers in 2025. Global Epidemiology, p.100257. HTP users: Mzhavanadze G, Stimson GV, Jerzyński T Global Heated Tobacco Product User Estimates, 2014–2024: Descriptive Surveillance Study Using Manufacturer Disclosures JMIR Public Health Surveill 2026;12:e88761.

This distinction is important. Continued nicotine use after smoking cessation should not automatically be regarded as treatment failure, especially if smoking has been eliminated. However, it should not be assumed to represent a full resolution of dependence. Persistent use of fast-delivery consumer nicotine products may maintain neuroadaptation, cue-driven use, and psychological attachment, particularly in the absence of structured tapering or cessation support. By contrast, prolonged use of licensed NRT can occur, but its abuse liability, reinforcement profile, and clinical context differ substantially from those of recreational consumer products (3, 13, 26).

## Case study: England—smoking declines, nicotine use rebounds

Despite longstanding declines in cigarette smoking, overall nicotine use in the United Kingdom has shown signs of resurgence, driven by rapid uptake of non-combustible nicotine products and a growing user base with no prior history of tobacco smoking.

The previously noted decline in combustible use has been paralleled by a sharp rise in non-combustible nicotine products. In 2023, 9.8% of adults in Great Britain, approximately 5.1 million people, reported current use of ENDS, with the highest prevalence among 16–24- year-olds (15.8%) (27). While many of these users are current or former smokers, a significant proportion are not. Data from a nationally representative study by Jackson et al., (28) indicate that by 2024, more than 1 million adults in England who had never smoked regularly were using ENDS, up from just 133,000 in 2021. Among never-smoking young adults aged 18–24, vaping prevalence reached 14% (7).

This pattern of uptake has contributed to a reversal in total nicotine use among young adults. An examination of the impact of disposable ENDS in England using data from the national Smoking Toolkit Study found that between May 2021 and May 2023, vaping prevalence among 18–24-year-olds rose from 9 to 29%, while smoking prevalence fell modestly from 25 to 21%. Overall nicotine use in this age group, defined as current smoking or vaping, increased from 28 to 35% over the two-year period (29), with a substantial proportion of this increase driven by individuals with no prior smoking history. It remains to be seen how the newly passed UK Tobacco and Vapes Bill (30) will influence these emerging patterns.

## Case study: US vaping increases, main source of nicotine shift

Recent US data indicate a marked shift in the preferred formats for nicotine consumption, with vaping/ENDS emerging as the dominant method among youth and young adults. According to a comprehensive study by Sun et al., the prevalence of vaping among US adolescents increased substantially between 2017 and 2021, surpassing traditional cigarette smoking in this age group (31). The rise in ENDS use has been driven by factors such as perceptions of reduced harm and the wide availability of vaping products.

Similarly, information from Smoke Free Capital highlights that vaping now accounts for most of the nicotine use among young populations, with a significant proportion of users having never smoked combustible cigarettes (32). Data from the National Institutes of Health (33) further underscore that non-combustible nicotine products like ENDS have become the primary source of nicotine for many new users, particularly in the 18–24 age group.

The New York Times illustrated this trend visually, showing that between 2017 and 2019, vaping overtook cigarette smoking as the most common nicotine delivery method among teenagers, with prevalence rates rising from approximately 11 to 27%. This rapid uptake has contributed to a reversal in traditional tobacco use patterns, with non-combustible products now playing a central role in nicotine consumption among young people (34).

Overall, these trends reflect a significant transformation in nicotine use formats, with vaping/e-cigarettes now constituting the primary mode of intake for a growing segment of the population, especially among youth and young adults who may not have previously engaged in tobacco smoking.

## Case study: Romania marketing-driven uptake of alternative nicotine products

Despite declining smoking rates across Europe, Romania continues to exhibit relatively high levels of combustible tobacco use alongside emerging uptake of alternative nicotine products, particularly heated tobacco products (HTPs). Recent estimates suggest that around 22–26% (35) of adults in Romania smoke, equivalent to over 4 million people (35), with substantially higher prevalence among men than women. This remains significantly higher than the ~10–11% (36) observed in England, highlighting persistent tobacco dependence within the population.

Unlike England, where the rise in nicotine use is largely driven by ENDS, the Romanian context highlights the role of regulatory gaps and direct marketing in shaping nicotine consumption patterns. Research by Sergiu Chirila et al. (37) demonstrates that HTPs have experienced rapid uptake among young adults, particularly in environments where advertising remains insufficiently regulated (37). Romanian legislation stipulates that indoor smoking is prohibited only if the tobacco is burned, leaving people free to use HTPs indoors (37).

Marketing plays a central role in this transition. Young adults are widely exposed to HTP promotion in retail environments, on social media, and through direct engagement with sales representatives (37). These strategies emphasise product appeal, such as modern design, technological features, and perceived reduced harm, while also normalising use within social spaces. Importantly, although participants often denied being influenced by marketing, their behaviours reflected strong engagement with these promotional strategies, suggesting implicit influence and self-rationalisation (37).

Perceptions of risk further contribute to uptake. While most participants acknowledged that smoking is harmful, many expressed ambivalence regarding the risks of HTPs, often viewing them as a “less harmful” alternative. Combined with strong peer and family influences, this supports experimentation and dual use, with many individuals continuing to smoke cigarettes alongside HTPs rather than switching completely (37). Moreover, doctors are seen as public health role models, and their smoking status can influence both their motivation and credibility in delivering smoking cessation.

## Case study: Japan and South Korea—rapid adoption of heated tobacco products and persistent dual use

East Asia provides a distinct perspective on the evolving nicotine landscape, particularly through the rapid uptake of HTPs in countries such as Japan and South Korea. These markets have experienced some of the fastest transitions from combustible cigarettes to alternative nicotine delivery systems globally, driven by technological innovation, strong commercial penetration, and, in Japan’s case, regulatory constraints on ENDS.

In Japan, where ENDS are effectively restricted, HTPs have become the dominant non-combustible alternative. Since their introduction in the mid-2010s, HTPs have captured a substantial share of the tobacco market, with estimates suggesting they account for over 30% of total tobacco sales (38, 39). This rapid uptake has coincided with a decline in cigarette consumption, often cited as a harm reduction success (40). However, this transition has not resulted in widespread nicotine cessation. Instead, a significant proportion of users engage in dual use, combining HTPs with conventional cigarettes.

Evidence suggests that approximately two-thirds of HTP users in Japan continue to smoke combustible cigarettes alongside HTP use (39, 41, 42). This pattern indicates that, for many individuals, HTPs function not as a complete substitute but as a complementary product used in situations where smoking is restricted or socially undesirable. As a result, overall nicotine exposure may be sustained rather than reduced, and the anticipated public health gains are partially diluted.

A similar pattern is observed in South Korea, where HTP uptake has also been rapid following their introduction. Within a few years, HTPs accounted for a significant share of the tobacco market (43). Notably, dual and poly-use are even more pronounced in this context, with studies reporting that over 90% of HTP users in South Korea also use other tobacco products, particularly cigarettes (44). This suggests that product switching is often incomplete and that nicotine dependence persists across multiple delivery formats.

These patterns highlight several important public health implications:

- Incomplete substitution: While HTPs may reduce exposure to some toxicants compared to cigarettes, their use alongside smoking limits potential health benefits.
- Sustained nicotine dependence: The availability of alternative products does not necessarily lead to cessation; instead, it may reinforce ongoing addiction through diversified consumption.
- Behavioural reinforcement: The coexistence of multiple product types allows users to maintain nicotine intake across different contexts, embedding dependence more deeply into daily routines.
- Policy complexity: Regulatory frameworks that focus on product substitution without explicitly addressing total nicotine cessation may inadvertently support long-term dependence.

The experiences of Japan and South Korea illustrate that shifts away from combustible tobacco do not automatically translate into reductions in overall nicotine use or dependence.

## Risk continuum and health effects of nicotine-containing products

Nicotine-containing products span a broad continuum of risk. At one end, combustible tobacco products remain the most harmful form of nicotine delivery because their disease burden is driven primarily by combustion-related toxicants, including carcinogens, carbon monoxide, fine particulates, and numerous chemicals implicated in cancer, cardiovascular disease, respiratory disease, and adverse reproductive outcomes (5, 8, 18). No safe threshold of smoking exposure has been established, and even low levels of cigarette consumption carry substantial risk, particularly for cardiovascular disease.

It is important to note that nicotine itself is not a direct carcinogen. It can cause confusion among clinicians due to its potential through tobacco use, to promote tumour growth and metastasis by increasing cell proliferation and blood vessel formation (45, 46). Nicotine also exerts diverse physiological effects, with the scale of any associated health risk shaped less by the molecule itself than by the route, dose, user population, and duration of exposure; nicotine acutely stimulates the cardiovascular system (47) through catecholamine release, increasing heart rate, blood pressure, and vascular resistance (18); chronic exposure may contribute to endothelial dysfunction and insulin resistance; whilst during pregnancy, nicotine impairs placental blood flow and has been associated with adverse birth outcomes including preterm delivery and low birth weight (18).

Non-combustible consumer nicotine products, including ENDS, HTPs, and oral nicotine products such as pouches, generally expose users to fewer combustion-related toxicants than cigarettes, especially when used as complete substitutes for smoking. However, they are not risk-free.

ENDS aerosols may contain carbonyl compounds, metals, and other harmful constituents; HTP aerosols contain nicotine and a range of toxicants, including carbonyls, volatile compounds, and tobacco-specific toxicants; and oral nicotine products may have adverse local effects and deliver high nicotine doses that sustain dependence (48–54, 97). Long-term data remain incomplete across several of these categories, and substantial heterogeneity between products complicates the interpretation of category-wide risk. Whilst some products are subject to quality standards, for example, under the EU Tobacco Products Directive (TPD; 2014/40/EU) and its UK implementation (Tobacco and Related Products Regulations; TRPR), the wide variety of devices and liquids available, manufactured with differing levels of oversight, results in substantial variability in aerosol composition, nicotine delivery, and exposure to harmful substances. This complicates the assessment of health risks across the category and becomes a diagnostic challenge for HCPs monitoring patient health.

Overall, however, studies have reported associations between use of these product categories and cardiovascular-relevant changes, including elevated heart rate and blood pressure, endothelial dysfunction, and reduced heart rate variability. Observational studies have also linked use with respiratory outcomes, including asthma exacerbations, particularly among youth, and chronic bronchitis symptoms (55–57). In addition, some analyses have reported higher myocardial infarction risk among users compared with non-users; however, these findings should be treated as associative rather than causal, given the potential for confounding and variation in exposure definitions (58, 98).

Second-hand exposure to these products is linked to asthma and bronchitis (56, 59). There is also evidence of an increased risk of periodontal disease and caries in vapers versus non-vapers (60). A recent nationwide South Korean cohort study found that e-cigarette use after smoking cessation was associated with higher lung cancer incidence and lung cancer-specific death than complete quitting, especially among high-risk former smokers. While observational, these findings suggest that continued e-cigarette use may attenuate some cancer-prevention benefits of complete cessation (61).

Snus contains tobacco and, in a pooled analysis of never-smoking men, current use was associated with increased all-cause, cardiovascular, and cancer mortality, with no single cancer type identified as the primary driver in the analysis of (62). The Byhamre analysis also links type 2 diabetes, obesity, and heart failure to this product, compared to never users. Nicotine pouches, often tobacco-free, are less studied but may deliver high nicotine doses. In-vitro studies indicate that some nicotine pouch extracts can cause cytotoxicity and pro-inflammatory responses in oral epithelial cells, and surveys report mouth and throat irritation among users (51). Some clinical evidence suggests that physical proximity during use is linked to severe periodontal disease via a toxic response in gingival epithelial cells (52) and that the pouches can impair the periodontal innate immune response (54). However, human clinical evidence on oral outcomes is limited to short-term studies, and high-quality, independent trials are still needed; animal data are also scant. A registered systematic review and network meta-analysis is underway to compare the oral side effects of non-combustible nicotine products, including pouches (63, 64).

Licensed nicotine replacement therapy occupies a different clinical category from consumer nicotine products. NRT is standardised, regulated, and intended as a therapeutic aid to support cessation through controlled dosing and defined clinical use. Its risks are well characterised and substantially lower than those of smoking (26, 65). Although prolonged use can occur, NRT differs from fast delivery consumer products in pharmacokinetics, behavioural reinforcement, regulatory oversight, and treatment intent (3, 13).

Taken together, the evidence supports a continuum of risk across nicotine-containing products, with combustible tobacco at the highest end and licensed medicinal nicotine products at the lowest (Figure 2). Non-combustible consumer products occupy intermediate positions that may represent reduced harm relative to smoking, but not harmlessness as evidence on long-term health effects is still emerging (66). A balanced clinical interpretation, therefore, needs to clearly distinguish among relative risk, residual risk, and persistence of dependence (5, 8).

**Figure 2:**
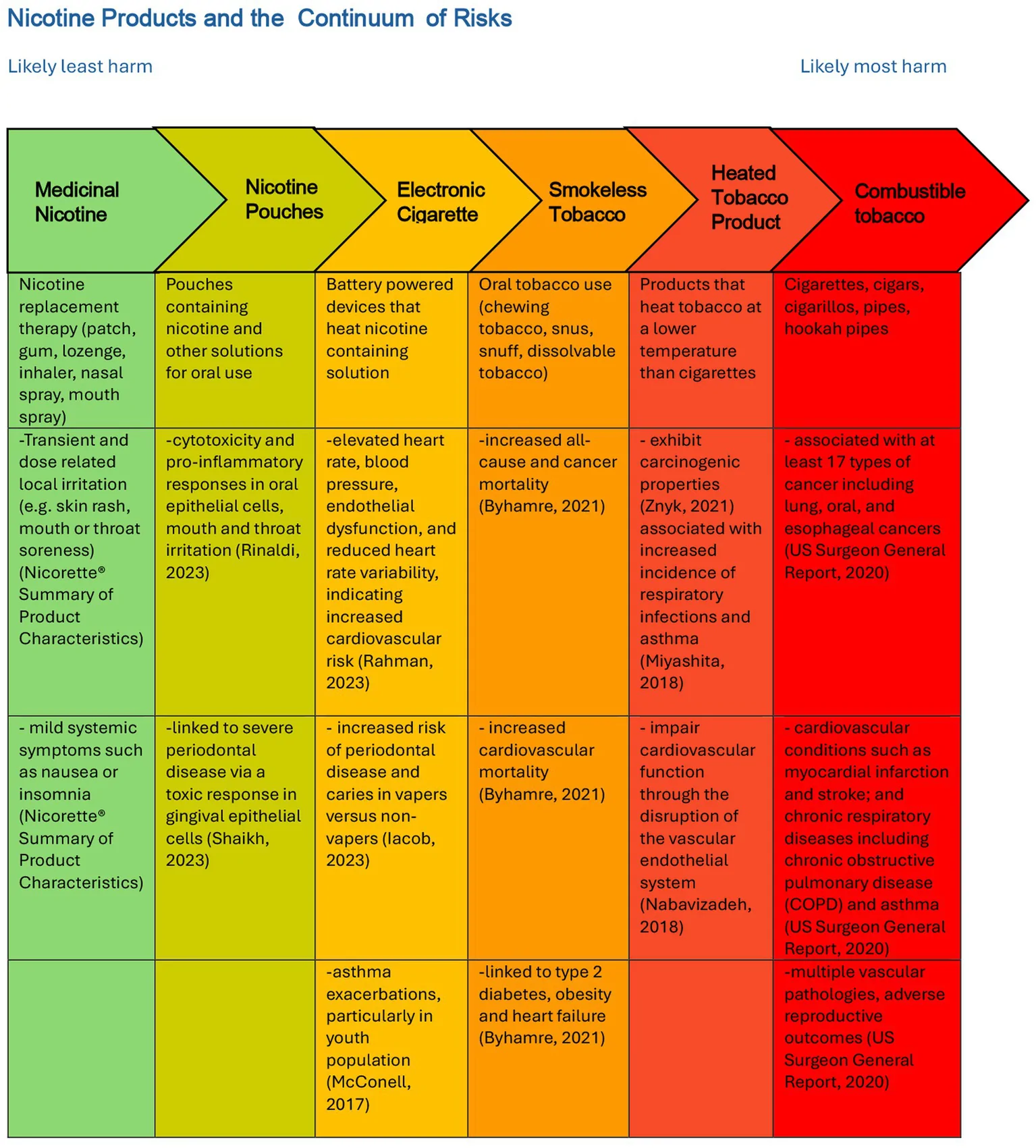
Adapted from Royal College of Physicians (5) and McNeill et al. (3), showing consensus that combustibles are the most harmful, NRT the least, with other non-combustible products occupying intermediate but not risk-free positions.

## Shifting nicotine use: dual use, vulnerable populations, and public health implications

Individuals who engage in the consumption of combustible cigarettes often concurrently use alternative nicotine delivery systems. This phenomenon, referred to as concurrent use (41), encompasses a diverse population of users who may employ two (‘dual use’) or more (‘poly use’) tobacco and nicotine products. Epidemiological data from the United States, derived from the Population Assessment of Tobacco and Health (PATH) Study, indicate that approximately 40% of nicotine users, encompassing both adult and youth demographics, engage in the use of multiple tobacco products, with the most prevalent combination being combustible cigarettes and ENDS (67). The International Tobacco Control Four Country Tobacco and Vaping (ITC 4CV) survey has reported varying rates of dual use among nicotine users (smokers and vapers), with the highest prevalence observed in Canada (13.0%), followed by England (12.4%), the United States (10.2%), and the lowest in Australia (2.6%) (68). In Japan, studies have estimated that approximately two-thirds of individuals who use HTP also smoke combustible cigarettes (39, 41, 42), while in South Korea, the concurrent use rate among HTP users was reported to be 96.2% in 2018 (44).

For some people who smoke, dual use may be an interim state as they transition away from cigarettes. Most dual users report using ENDS to quit or cut down on cigarette smoking (69). However, the process of fully transitioning from cigarettes to an alternative product (or complete abstinence from tobacco) may be a long-term process. Thus, dual and poly use may be an interim state for people attempting to quit smoking cigarettes. Conversely, if alternative products serve as complementary products, concurrent use may sustain smoking cigarettes (44). Current evidence suggests dual users are more likely to attempt to quit smoking than those who smoke exclusively (70).

Engaging dual and poly users in smoking cessation programmes is beneficial, as they may already be motivated to quit. However, fully transitioning from combustible cigarettes to alternative products or achieving complete abstinence from all tobacco and nicotine products is challenging. Many dual and poly users prefer a stepwise approach, quitting one product at a time, rather than attempting to quit all products simultaneously (71). This approach can be more manageable and may increase the likelihood of successful cessation.

Further research is needed to evaluate the effectiveness of different cessation strategies, such as simultaneous versus stepwise cessation, specifically for dual and poly users. Understanding the unique challenges and motivations of these users can help develop more tailored and effective interventions to support their smoking-cessation efforts.

More broadly, non-combustible nicotine products have reshaped nicotine use across populations. Still, high rates of dual use (combining non-combustible nicotine products with continued smoking) may dilute their public health benefit, particularly when they do not lead to cessation or substantial reduction (72). A recent systematic review confirms that dual users are less likely to achieve smoking or nicotine abstinence than exclusive smokers or exclusive ENDS users, with many reverting to cigarette smoking, prolonging exposure to toxicants (73). Another study (99) also found that dual use was associated with higher point estimates for odds of disease compared with cigarettes. Among never smokers, sustained use introduces new pathways to dependence (6, 74), and among switchers, many remain chronically dependent on nicotine, further complicating harm reduction claims.

Vulnerable populations, including those with mental health conditions, neurodevelopmental traits, and socioeconomic disadvantage, may derive disproportionate benefit from non-combustible nicotine products, especially if they struggle with traditional cessation. However, these groups also face barriers to accessing structured support. Non-combustible nicotine products’ use may become entrenched in the absence of medically supervised alternatives (75).

These shifts in usage patterns create challenges not only for consumers but also for clinicians and public health professionals, who must navigate variable policy signals, evolving terminologies, and patient assumptions about product risk and purpose (2). Such assumptions may arise from non-combustible nicotine products marketed as recreational products, often with little alignment to existing cessation infrastructure (76). As a result, individuals most in need of support to address their use may be least likely to receive it, either because they are not targeted by public health campaigns, or because they do not identify as needing cessation (77).

In summary, non-combustible nicotine products present both opportunities and challenges for public health. Realising their benefit requires more than availability; it depends on uptake patterns, integration with cessation pathways, and the ability to prevent new dependencies in populations who would otherwise have remained nicotine-free.

## Cessation pathways

Motivation to quit remains relatively high: in the United States, about two-thirds of smokers report they want to stop (78). The global figure reported by the World Health Organisation for those who want to quit is at 60% (79).

Historically, most smokers attempted to quit smoking unaided (‘cold turkey’). Population data from England suggest this has declined as licensed pharmacotherapies and, more recently, vaping products have become established aids, with a growing proportion of quit attempts now involving support (3).

There is a substantial intention among users of non-cigarette nicotine products to quit. Specifically, data from the Truth Initiative indicates that more than half (54.2%) of current ENDS users express a desire to stop using these products, reflecting a significant motivation to cease use (80). Similarly, a recent survey highlighted on the South Danish University (SDU) website reports that four out of five (80%) young users of nicotine pouches or similar products want to quit, demonstrating high levels of quitting intention within this demographic (SDU week 182,025).

In summary, data suggest that despite the rise in use of non-cigarette nicotine products, a large proportion of users are aware of potential harm or wish to discontinue their consumption.

Nicotine Replacement Therapy (NRT) remains the most widely used and widely studied pharmacological aid for achieving smoking and nicotine cessation within a structured quit attempt. Approved forms include patches, gum, lozenges, sprays, and inhalators. The patch, gum, lozenge, and mouthspray formats are included on the WHO Essential Medicines List. These products deliver nicotine at controlled, therapeutic levels that mitigate withdrawal while reducing reinforcement. Using a combination of behavioural support and medication might typically increase the chances of quitting smoking by 70 to 100% compared to their chance of success if they just received brief advice or support (81). Efficacy varies by format, ranging from Risk Ratio (RR) 1.49 (95% CI 1.40 to 1.60) for gum to 1.64 (95% CI 1.53 to 1.75) for patches, and up to 2.48 (95% CI 1.24 to 4.94) for oral spray, though higher estimates often come from fewer trials (65). Combination NRT (e.g., patch plus oral form) significantly improves quit rates compared with monotherapy and is recommended for more dependent users. A recent Cochrane review reported an RR of 1.27 (95% CI 1.17 to 1.37) for combination NRT vs. single-form (82).

NRT is generally well tolerated, with side effects typically limited to local irritation (e.g., skin rash, mouth or throat soreness) or mild systemic symptoms such as nausea or insomnia. These are usually transient and dose-related, and unlike recreational nicotine products, NRT’s risks are well characterised and substantially outweighed by its cessation benefits.

Non-combustible nicotine products (including ENDS and nicotine pouches) are frequently used by individuals trying to quit smoking. Evidence supports their short-term utility, with high-certainty findings that nicotine ENDS outperform NRT for smoking cessation in clinical trials (4). However, these trials typically track the endpoint of abstinence from combustible tobacco (“smoking cessation” in the sense defined above), not nicotine, and prolonged non-combustible nicotine product use is common. UK population data show that long-term ENDS use among ex-smokers is nearly nine times more prevalent than long-term NRT use (83). More recent prospective cohort evidence further highlights the importance of distinguishing short-term smoking abstinence from longer-term relapse risk. In the American Cancer Society Cancer Prevention Study-3, Westmaas et al. (96) compared ENDS and NRT use among adults who had already quit smoking or were smoking at baseline. Among those who had already quit, past exclusive ENDS use was associated with a higher risk of relapse to cigarette smoking than past exclusive NRT use, both in unadjusted analyses (11.2% vs. 3.9%; RR 2.90, 95% CI 2.12–3.98) and after multivariable adjustment (RR 2.09, 95% CI 1.49–2.92).

Cytisine and Varenicline—Cytisine and varenicline are partial agonists at nicotinic receptors. They reduce cravings and withdrawal symptoms while blocking nicotine’s reinforcing effects. In clinical studies, with behavioural support, varenicline has been shown to more than double quit smoking rates compared to placebo (RR 2.32), and to outperform NRT monotherapy (RR 1.25), although it appears broadly comparable to combination NRT (RR 1.02) (84). Cytisine has demonstrated superior efficacy to single agent NRT for smokers in two randomised controlled trials, with a 2024 meta-analysis reporting a pooled risk ratio of 1.39 in favour of cytisine. When compared with placebo, cytisine more than doubles the chance of successful smoking cessation (RR 2.25) (85), confirmed in a pivotal Phase 3 trial, ORCA-2 (86).

Bupropion, an atypical antidepressant that inhibits dopamine and norepinephrine reuptake, is also recommended as a first-line cessation aid in many clinical guidelines. Though less effective than varenicline or combination NRT, it has been shown to improve quit rates compared with placebo or no pharmacological treatment (RR 1.60, 95% CI 1.49 to 1.72) when combined with behavioural support (87).

Behavioural and Digital Interventions - Whether using NRT or non-nicotine options, the value of combining behavioural and pharmacological strategies for smoking cessation is clear, with evidence consistently showing higher quit rates when behavioural techniques are added to pharmacotherapy (10, 65, 84).

Cognitive behavioural therapy (CBT) addresses both the physiological and psychological drivers of dependence and is the most established framework for cessation support. It helps individuals identify high-risk situations, recognise and reframe triggers, challenge unhelpful beliefs about nicotine use, and develop coping strategies to manage cravings. This approach reduces relapse risk and enhances self-efficacy, making CBT-informed counselling central to most evidence-based cessation programmes (65, 84).

Digital delivery (including quit lines, text messaging, and web-based tools) further extends reach and effectiveness, particularly when underpinned by CBT principles (10, 88). Among these, text messaging programmes have the strongest evidence, significantly increasing quit rates compared to minimal support (RR 1.54, 95% CI 1.19 to 2.00) (88). Other tools such as quit apps and AI-based coaching show promise for increasing engagement and personalisation, with emerging evidence from a recent systematic review of conversational AI showing significantly higher quit rates (RR 1.29, 95% CI 1.13–1.46) (89), though results should be interpreted with caution due to the early, heterogeneous evidence base.

Online communities and peer-based social support are also emerging as effective adjuncts, particularly among younger users or those with limited access to in-person services. Evidence from a systematic review of social media interventions indicates that platforms such as Facebook, Twitter, and WhatsApp can facilitate engagement, sustain motivation, and contribute to improved quit outcomes, including increased quit attempts and reduced relapse (90). These tools provide scalable, cost-effective ways to deliver tailored feedback and reinforce quitting behaviours in real time.

## Smoking cessation to nicotine cessation: why the endpoint matters

For decades, cessation policy and treatment were built primarily around combustible tobacco. In that context, quitting smoking often meant quitting nicotine. That assumption is less reliable in today’s product environment, where nicotine use may continue through ENDS, HTPs, pouches, or medicinal products after cessation of combustible tobacco products. Smoking cessation and nicotine cessation should therefore be treated as distinct, though related, outcomes (3, 10).

Smoking cessation remains a major public health gain and should not be minimised. For people who smoke, complete switching away from combustible tobacco may substantially reduce exposure to the toxicants most responsible for smoking-related disease (4, 5). Harm reduction, therefore, retains an important role, particularly for individuals who are unable or unwilling to stop smoking immediately. However, smoking abstinence alone does not necessarily resolve nicotine dependence, and some users may remain dependent for prolonged periods without a clear route toward full cessation (9, 24).

Nicotine cessation refers to the discontinuation of all nicotine use, regardless of product type. This endpoint may matter for several reasons: it eliminates ongoing pharmacological dependence, reduces cue-driven and habitual use, removes uncertainty associated with long-term exposure to newer products, and avoids complications of prolonged dual use or repeated switching between product types (8, 18). In that sense, nicotine abstinence represents a broader and more complete endpoint than smoking abstinence alone.

This distinction should be reflected in both terminology and care pathways. Switching should not be conflated with quitting, and reduced harm should not be conflated with resolution of dependence. A more precise framework would recognise harm reduction as one possible route within a cessation journey, while retaining complete nicotine abstinence as the preferred long-term destination where achievable.

Reduction without a clear cessation endpoint may leave nicotine dependence largely intact and can be accompanied by compensatory behaviour, prolonged dual use, or indefinite maintenance on alternative products. For this reason, reduction should be understood as a possible stage within a cessation pathway rather than as the preferred endpoint in itself (72, 73, 91). The central position of this review is therefore not that harm reduction lacks value, but that its value should be assessed in relation to what follows it. When it becomes a bridge to complete cessation, it may be clinically useful. When it becomes a durable substitute for nicotine abstinence without a clear endpoint, it risks entrenching dependence and blurring the meaning of successful cessation.

## Clinical implications for healthcare professionals

Healthcare professionals (HCPs) are uniquely positioned to support complete nicotine cessation, particularly as the nicotine product landscape becomes more complex and fragmented. While most clinical guidelines remain focused on smoking cessation, nicotine users who do not smoke, including long-term users of ENDS, HTPs, pouches, other non-combustible nicotine products, and to some degree even NRT, are not uncommon. Dependency may continue, often without access to clinical guidance or cessation services (3, 10). Although formal evidence for treating non-smoking nicotine dependence is limited, HCPs can play a vital anticipatory role, helping users explore routes out of addiction, correcting misperceptions, and supporting long-term abstinence as a credible goal.

To meet this challenge, HCPs, especially frontline professionals such as nurses and pharmacists, must broaden their remit from tobacco cessation to nicotine cessation. This means actively assessing all forms of nicotine use during consultations, offering guidance on complete cessation - not just switching between nicotine-containing products - and referring patients to evidence-based support such as behavioural counselling and pharmacotherapy. Brief interventions can use motivational interviewing, the 5As framework, or brief advice models to clarify whether the patient’s goal is smoking cessation, nicotine cessation, or staged reduction (92). Follow-up should assess not only smoking abstinence but also ongoing nicotine use, dual use, withdrawal symptoms, relapse risk, and readiness to progress toward complete nicotine abstinence. HCP’s can also provide education on the distinction between reduced toxicant exposure and resolution of dependence, helping patients understand harm reduction as a possible step within a broader cessation plan rather than as an inevitable endpoint.

Effective engagement can also help dispel common misperceptions. Terms such as ‘vaping,’ ‘smoke-free,’ and ‘alternative products’ can frame novel nicotine formats as inherently safer, distorting public and clinical perception of their risks (100), which may reinforce the belief that continued vaping is synonymous with quitting. Addressing this further, HCPs are well placed to identify patients experiencing transferred dependency, particularly those who feel conflicted between having quit tobacco, yet who continue to rely on nicotine (9, 24).

Clinical guidelines and training materials should reflect this broader mandate. They should clearly differentiate between harm reduction, reductions to quit, and complete abstinence, and equip HCPs with the tools to discuss nicotine use in practical, non-judgmental terms (3, 10).

By expanding their focus from tobacco cessation to include broader nicotine use, HCPs can help bridge an emerging clinical gap. While most cessation tools were developed for smokers, many can still support users seeking to treat the same nicotine dependence from other formats, and licensed indications are starting to reflect this in places such as the UK, Australia, and New Zealand. Updating clinical training and service pathways to reflect this evolving need will ensure that cessation care remains relevant, initiative-taking, and inclusive, even as formal evidence and regulation continue to develop. Empowering HCPs in this way enables health systems to deliver consistent, forward-looking care that supports complete nicotine abstinence as a realistic and supported goal.

## Research priorities

The evidence base for complete nicotine cessation remains less developed than that for smoking cessation, particularly among users whose dependence arises primarily through non-combustible products. Several priorities follow from this gap.

First, more long-term data are needed for exclusive and dual users of non-combustible nicotine products, including emerging formats such as high-concentration disposable ENDS and nicotine pouches. Chronic exposure patterns, dose trajectories, and long-term cardiometabolic, respiratory, and oral health effects remain incompletely understood (8, 64, 93).

Second, more research is required on the psychosocial drivers of sustained nicotine use among non-smokers and ex-smokers, including ambivalence, dependence identity, and unmet desire to quit. Qualitative and longitudinal studies may help clarify why some users remain on non-combustible nicotine products for prolonged periods despite intending to stop (9, 24).

Third, clinical studies should evaluate how established cessation tools including NRT, cytisine, varenicline, behavioural support, and digital interventions can be adapted for vaping cessation and broader nicotine cessation. The evidence base for vaping cessation is beginning to develop: a recent systematic review and meta-analysis of RCTs found that pharmacological, educational, and digital interventions significantly increased short-term abstinence rates, though the certainty of the evidence remains limited (94). Existing clinical guidelines, however, focus almost exclusively on smoking; further controlled trials and implementation studies are needed to evaluate strategies that address dependency beyond combustion (3). Future studies should also standardise outcome measures for nicotine cessation. Trials should distinguish between smoking abstinence, nicotine abstinence, dual use, product switching, reduction in dependence severity, and relapse to combustible tobacco. Comparative-effectiveness studies are needed to determine which combinations of behavioural support, pharmacotherapy, digital tools, and structured follow-up by healthcare professionals are most effective for different user groups, including exclusive ENDS users, pouch users, dual users, and people with high dependence or comorbid mental health conditions. This should include models delivered by nurses, pharmacists, physicians, respiratory specialists, behavioural counsellors, and other frontline healthcare professionals.

Fourth, communication research should examine how different framings of risk, quitting, and harm reduction affect understanding, motivation, and behaviour across groups such as youth, dual users, never smokers, and long-term users of non-combustible products (10, 95).

Finally, population-level modelling should evolve to reflect patterns of nicotine use, not just smoking prevalence. Policies that incentivise smoking reduction without addressing nicotine cessation may unintentionally sustain or shift dependency. Levy et al., (72) propose a behavioural framework to model transitions between product use states, including dual use, switching, and abstinence. Building on this, future forecasting tools should incorporate patterns of nicotine use, not just smoking, to estimate public health outcomes more accurately.

## Limitations

This review has several limitations. First, it is a narrative rather than systematic review and therefore reflects selective synthesis rather than exhaustive evidence retrieval. Second, the evidence base is uneven across product categories: combustible tobacco is extensively studied, whereas long-term data for several non-combustible products, particularly newer pouches and rapidly evolving device formats, remain limited. Third, many cessation studies continue to use smoking abstinence rather than nicotine abstinence as the primary endpoint, which complicates interpretation when continued nicotine use is common after cigarette cessation.

In addition, product categories are heterogeneous. Differences in formulation, delivery kinetics, regulation, user behaviour, and market evolution make it difficult to draw simple conclusions across broad categories such as “e-cigarettes” or “oral nicotine products” (2, 8). Terminology in this field also remains unsettled. Concepts such as quitting, switching, harm reduction, maintenance, and transferred dependence are not always used consistently across studies or policy frameworks (3, 10).

These limitations reinforce the need for caution in interpretation. The position advanced in this review should therefore be understood as a clinically and public-health oriented interpretation of an evolving evidence base, rather than as a claim that all uncertainties have been resolved.

## Conclusion

The diversification of nicotine products has changed the meaning of cessation. Smoking abstinence remains a major clinical and public health gain, and for some people who smoke, non-combustible products may reduce exposure to the toxicants most responsible for smoking related disease (4, 5). However, reduced exposure to toxicants should not be conflated with the resolution of nicotine dependence. In an environment where nicotine use may persist through ENDS, HTPs, nicotine pouches, or other formats, smoking cessation and nicotine cessation are no longer interchangeable endpoints.

This review argues that harm reduction may be appropriate as part of a stepwise pathway away from combustible tobacco, but that complete nicotine abstinence should remain the preferred long-term clinical and public health goal. While harm reduction typically involves an open-ended substitution with no strategy to stop nicotine, a Total Quit framework may utilise a planned, temporary phase of dual use or nicotine substitution as a structured pathway toward complete abstinence. The summary of the pros and cons of both approaches (“*Total Quit*” vs. harm reduction) is presented in Table 1. This position does not deny the value of transitional strategies. Rather, it seeks to reestablish clarity: switching may reduce harm, but only cessation ends dependence.

**Table 1:** A side-by-side comparison of the “*Total Quit*” and harm reduction approaches to nicotine addiction.

Approach	Pros	Cons/Challenges
“*Total Quit*” *(Complete Nicotine Abstinence)*	Fully resolves dependence: Ends the underlying pharmacological addiction, neuroadaptation, and cue-driven behavioural loops. Eliminates residual risk: Removes the uncertain long-term health risks associated with alternative non-combustible products. Avoids dual use: Bypasses the complications of prolonged concurrent use or indefinite product switching. Optimal endpoint: Represents the safest and most complete long-term clinical and public health goal.	High difficulty: Harder to achieve outright, particularly for highly dependent users or those without access to structured behavioural and pharmacological support. Overwhelming for some individuals: Many users prefer a stepwise approach; quitting all nicotine simultaneously can be overwhelming and deter quit attempts. Risk to vulnerable groups: May leave individuals who struggle with traditional cessation methods without transitional support.
“Harm Reduction” *(*e.g.*, Transitioning to ENDS, HTPs, or Pouches)*	Toxicant reduction: Substantially lowers exposure to combustion-related carcinogens, carbon monoxide, and particulates when fully replacing cigarettes. Stepwise bridge: Serves as a practical, transitional pathway for individuals unwilling or unable to stop smoking immediately. High short-term utility: Vaping has been shown in some trials to outperform traditional Nicotine Replacement Therapy (NRT) for short-term smoking cessation. Aids vulnerable populations: Can provide disproportionate benefits to groups (e.g., those with mental health conditions) who struggle with traditional abstinence.	Sustains addiction: Does not resolve nicotine dependence, often resulting in “transferred dependence” to the new product. Risk of “Dual Use”: Many users continue smoking cigarettes concurrently, which dilutes health benefits and prolongs toxic health exposures. Not risk-free: Continued use poses intermediate health risks, including cardiovascular strain, respiratory irritation, and potential periodontal issues. Lack of clinical structure: Unlike licensed NRT, consumer products lack formal guidance or structured tapering, often leading to indefinite maintenance. Youth initiation: Creates new pathways for nicotine dependence among young people and never-smokers.

A modern cessation framework should therefore distinguish more clearly between smoking abstinence and nicotine abstinence, support users across all product types, and provide realistic pathways toward full cessation. In that context, *Total Quit* is best understood as the point at which nicotine dependence has been fully resolved, an outcome that remains clinically coherent, evidence-informed, and relevant to both public health and patient care.

## Footnotes

1 https://www.sdu.dk/en/sif/ugens_tal/ut_18_fire_ud_af_fem_unge_vil_droppe_nikotinposerne. Accessed 27 October 2025

2
https://ourworldindata.org/smoking
